# READRetro web: A user-friendly platform for predicting plant natural product biosynthesis

**DOI:** 10.1016/j.mocell.2025.100235

**Published:** 2025-06-02

**Authors:** Yejin Kwak, Taein Kim, Sang-Gyu Kim, Jeongbin Park

**Affiliations:** 1 Medical Research Institute, Pusan National University, Yangsan, Republic of Korea; 2 Department of Biological Sciences, KAIST, Daejeon, Korea; 3 School of Biomedical Convergence Engineering, Pusan National University, Yangsan, Republic of Korea

**Keywords:** Computational tools, Natural products, READRetro, Retrosynthesis, Web platform

## Abstract

Natural products (NPs), a fundamental class of bioactive molecules with broad applicability, are valuable sources in pharmaceutical research and drug discovery. Despite their significance, the large-scale production of NPs is often limited by their availability and scalability, requiring alternative approaches such as metabolic engineering or biosynthesis. To identify ideal pathways for the mass production of NPs, deep learning-based retrosynthesis models have been recently developed. Such models accelerate NP discovery; however, these tools are often not easy to use for researchers with a limited computational background, because they require complex environment configurations, command-line interfaces, and substantial computational resources. Here, we introduce READRetro web, a user-friendly web platform that integrates the READRetro machine learning (ML) model for retrosynthesis prediction. Based on modern web technologies, our web platform provides a fast and responsive user experience. READRetro Web bridges the gap between advanced ML-driven retrosynthesis and practical research workflows, making retrosynthesis prediction accessible to a broader range of researchers. Our platform demonstrates high predictive accuracy and computational efficiency, offering well-organized results to facilitate NP retrosynthetic pathway design. READRetro Web is freely accessible via https://readretro.net.

## INTRODUCTION

Natural products (NPs) have long been recognized as valuable molecular sources across diverse applications, including foods and chemical fragments, and have played a particularly significant role in the pharmaceutical industry. Over 30% of U.S. Food and Drug Administration (FDA)-approved small-molecule drugs are derived from NPs or their derivatives, highlighting their importance in drug discovery and development (Newman and Cragg, 2020). However, large-scale production of NPs remains challenging, as they are predominantly extracted from native host organisms, which often leads to limitations in availability and scalability (Courdavault et al., 2021). To address this, biosynthesis or semisynthesis through metabolic engineering has emerged as a promising solution, enabling more efficient and sustainable production of NPs. The success of such approaches heavily depends on a comprehensive understanding of the biosynthetic pathways within native hosts, as these pathways hold the key to unlocking large-scale production and further applications of NPs (Nett et al., 2020). Advances in computational tools, such as retrosynthesis models, have further transformed the field by enabling the systematic design of biosynthetic pathways, thus accelerating NP discovery and their integration into modern medicine (Finnigan et al., 2021, Ishida et al., 2022, Kim et al., 2024, Koch et al., 2020, Levin et al., 2022, Zheng et al., 2022).

Despite these advancements, the application of such tools remains challenging for researchers with limited computational expertise, as they often require configuring complex environments, using command-line interfaces, and managing local computational resources. For instance, programs like retropathRL demand a command-line-based environment and specialized knowledge in Python setup and model training due to their reliance on complex reinforcement learning algorithms (Koch et al., 2020). To overcome these barriers, we developed the READRetro web platform, which integrates the READRetro ML model with an intuitive, web-based interface (Kim et al., 2024). This platform simplifies the process of retrosynthesis prediction by offering an intuitive interface and well-organized results, allowing researchers to efficiently explore biosynthetic pathways without requiring extensive computational knowledge.

The READRetro web platform leverages modern web technologies to ensure scalability, accessibility, and responsiveness, effectively bridging the gap between cutting-edge ML tools and practical research workflows. Designed with modern web technologies, the READRetro website ensures an intuitive user experience while handling complex computational tasks. The frontend, built using Svelte (https://svelte.dev), Flowbite-Svelte (https://flowbite-svelte.com), and TailwindCSS (https://tailwindcss.com), offers a responsive and intuitive interface. Svelte-optimized compilation reduced runtime overhead, enabling faster load times and a smoother user experience. TailwindCSS enhanced visual consistency and performance by efficiently managing styles through utility classes, while Flowbite-Svelte provided cohesive and functional components like buttons and input fields, further simplifying user interactions.

On the backend side, FastAPI (https://fastapi.tiangolo.com) powered the platform with its robust asynchronous framework, ensuring efficient, large-scale data processing and rapid response times. By leveraging Python’s asynchronous capabilities, FastAPI handled concurrent requests and seamlessly integrated the READRetro model as application programming interface (API) endpoints, allowing users to perform retrosynthesis predictions effortlessly. Docker was employed to containerize the platform, ensuring stable performance across various environments and guaranteeing consistent deployment and reliability. This streamlined integration of advanced technologies enabled the READRetro website to deliver accurate predictions and well-organized results, making state-of-the-art retrosynthesis accessible to researchers with minimal computational expertise.

## MAIN BODY

### Main Interface of READRetro web

**Fig. 1:**
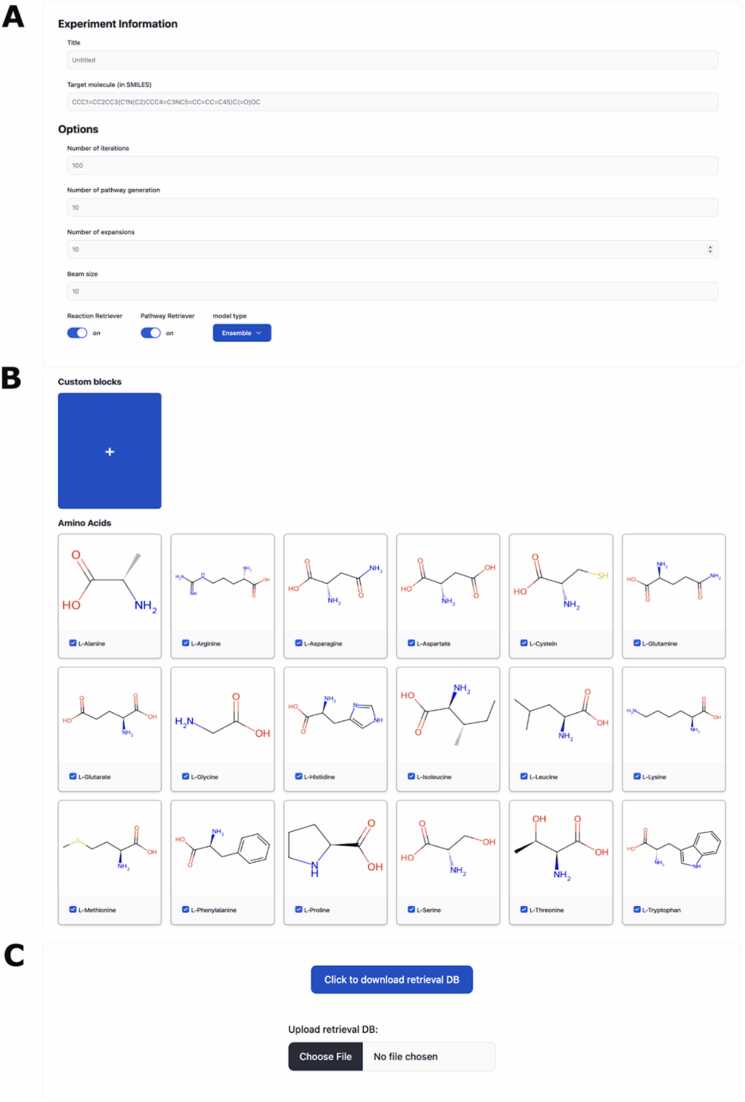
Overview of READRetro web interface and key functional tabs. (A) The "Arguments" tab provides users with control over key model parameters, allowing fine-tuning of the number of iterations, pathway generation, expansions, beam size, retriever type, and model type to optimize computational efficiency and prediction accuracy. (B) The "Building Blocks" tab includes 40 essential biosynthetic precursors, enabling users to construct pathways efficiently. A custom blocks section allows for the addition of unique building blocks via SMILES strings, facilitating the exploration of novel biosynthetic routes. (C) The "Retrieval DB" tab enables users to modify the database used for pathway prediction by adding or excluding specific reactions or pathways, supporting both focused and exploratory analyses of biosynthetic networks.

The platform was designed with 3 main goals based on modern web technologies: (1) to optimize the number of concurrent READRetro instances running on a single web server, (2) to maximize performance to address specific user needs, and (3) to deliver clear visualizations of the model’s results. To achieve optimal model performance, READRetro provides 3 interactive tabs—"Arguments", "Building Blocks", and "Retrieval DB"—that allow users to control key aspects of model operation (Fig. 1).

In the Arguments tab, users can adjust critical parameters, including the number of iterations, pathway generation, expansions, beam size, retriever type, and model type (Fig. 1A and Table 1). This customization enables users to fine-tune the model's functionality according to their requirements, supporting precise optimization of the READRetro model to enhance computational efficiency and prediction accuracy.

**Table 1:** Summary of READRetro parameters available in the Arguments tab, detailing adjustable settings that influence model performance and pathway retrieval

Parameter	Description
Number of iterations	The maximum depth of pathway exploration
Pathway generation	number of displayed alternative biosynthetic routes
Number of expansions	number of the reactants generated at each retrosynthesis step
Beam size	The number of candidate pathways retained per beam search iteration
Retriever types	(Reaction retriever, pathway retriever) whether to integrate known metabolic reactions
Model type	Ensemble, Retroformer, Graph2SMILES, and retriever only

The Building Blocks tab was implemented to include 40 essential building blocks, representing common precursors in NP biosynthesis pathways. This allows users to efficiently select relevant components for pathway construction (Fig. 1B). A custom blocks section was also provided, enabling users to add unique building blocks using SMILES strings, thereby facilitating tailored exploration of novel pathways.

The Retrieval DB tab was developed to give users control over the retrieval database utilized for pathway prediction (Fig. 1C). By allowing modifications—such as adding or excluding specific reactions or pathways—this feature enables researchers to either concentrate on established biosynthetic routes or investigate unexplored reactions by excluding known pathways, supporting focused and versatile pathway exploration.

### Server-Side Implementation

**Fig. 2:**
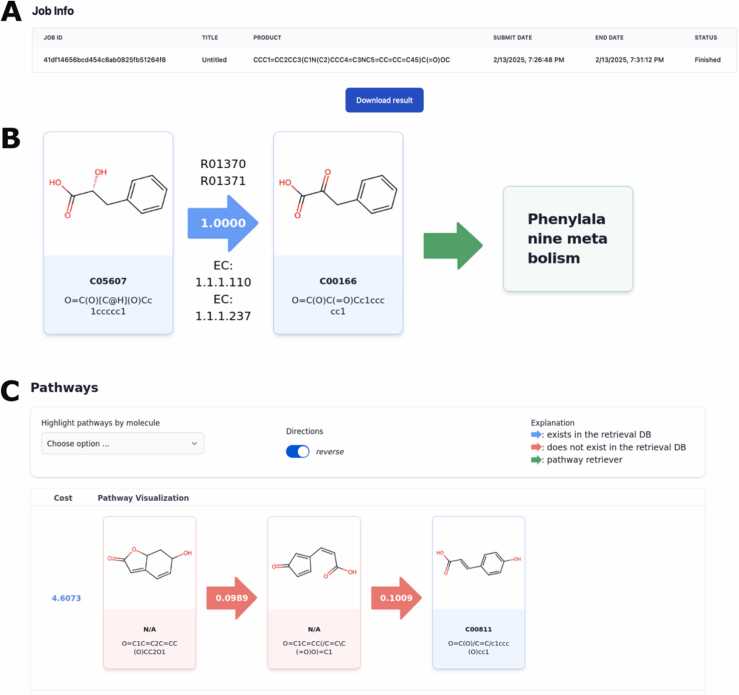
Task management and result visualization features of READRetro web. (A) The job ID infrastructure enables efficient task management by assigning unique job IDs to each READRetro instance. (B) The informative design of the result page integrates interactive cards and arrows to visually represent pathway relationships. Arrows are color-coded to indicate retrosynthesis availability, while enzyme commission (EC) numbers, kyoto encyclopedia of genes and genomes (KEGG) pathway information, and single-step scores are displayed for enhanced interpretability. (C) The result page delivers a comprehensive visualization of READRetro’s output, allowing users to highlight compounds, adjust pathway direction, and save results for later analysis.

To maximize concurrent READRetro instances on a server, a Docker-enabled Job ID infrastructure was implemented (Fig. 2A). After user-defined parameters are provided, READRetro initiates execution, with each instance assigned a unique Job ID for further tracking. Task distribution is managed by Celery, in tandem with Redis as an asynchronous task queue manager, while PostgreSQL stores Job IDs, metadata, and the results from the READRetro model. Docker containers encapsulate each component, ensuring environment consistency and simplified deployment. This robust, Docker-enabled infrastructure supports efficient processing and reliable job management, enabling seamless tracking and monitoring of tasks across the system through Job IDs.

### Visualization

The result page was developed to deliver intuitive visualization and efficiently summarize the model’s outputs (Fig. 2BC). The arrows between compounds indicate multiple layers of information as follows (Fig. 2B). The arrow colors were coded to indicate single-step retrosynthesis availability in the retrieval database, with green arrows specifically marking pathway retriever data. Furthermore, enzyme commission numbers, which classify enzymes based on the reactions they catalyze, and kyoto encyclopedia of genes and genomes (KEGG) pathway information, identifying biological pathways related to the compounds, were displayed above and below each arrow. These features help users to access to relevant entries in the BRENDA Enzyme Database and KEGG pathway by clicking on the links (Chang et al., 2021, Kanehisa et al., 2021). Additionally, single-step scores were displayed as numerical values on the arrows (Kim et al., 2024). The representation of compounds was based on cards containing detailed information, including SMILES notation, KEGG entries, and structural diagrams. Also, clicking the KEGG entry redirects users to the corresponding KEGG COMPOUND Database page. To enhance user experience, the result page additionally supports the highlighting of specific compounds, changing pathway direction, and comprehensive result saving for later access.

Preceding each pathway, cost values used for ranking pathways are displayed (Fig. 2C). The cost values are computed by applying boundary constraints (1e-3 ≤ score ≤ 1.0) to single-step scores, followed by logarithmic transformation, and subsequently aggregating these transformed values to ensure computational stability. The cost values directly reflect the internal ranking methodology employed during model inference (Kim et al. 2024).

## CONCLUSION

The development of web platforms for deep learning-based retrosynthesis represents a transformative step toward making these advanced computational tools more accessible and practical for researchers across diverse fields (Delépine et al., 2018, Kim et al., 2024, Zheng et al., 2022). However, despite the potential of such platforms, existing tools often face critical limitations that hinder their usability and impact. For instance, RetroPath 2.0, while technically accessible, remains largely inoperable due to maintenance challenges (Delépine et al., 2018), and BioNavi-NP, despite being functional, suffers from significant performance bottlenecks that restrict its practical utility (Kim et al., 2024). The introduction of the READRetro website addresses many of these challenges by combining the high-performance READRetro model with a user-friendly interface designed to deliver accurate and actionable retrosynthesis predictions. By leveraging advanced web technologies, the platform ensures seamless integration of complex computational tasks while maintaining accessibility for users with varying levels of computational expertise. This innovation extends beyond traditional retrosynthesis to uncover alternative biosynthetic pathways, optimizing metabolic engineering strategies, and supporting applications in diverse areas such as drug discovery, fragrance development, and bioengineering. Through ongoing refinement, READRetro web is poised to play a pivotal role in advancing NP biosynthesis, bridging computational innovations with real-world applications. We acknowledge the importance of batch processing for high-throughput applications. In this regard, we offer public web APIs (https://readretro.net/api/v1/docs) and a downloadable READRetro model with comprehensive documentation available on the Read the Docs platform (https://readretro.readthedocs.io).

## CRediT authorship contribution statement

**Yejin Kwak:** Writing – original draft, Software, Methodology, Conceptualization. **Jeongbin Park:** Writing – review & editing, Supervision, Software, Methodology, Funding acquisition, Conceptualization. **Taein Kim:** Writing – review & editing, Writing – original draft, Conceptualization. **Sang-Gyu Kim:** Writing – review & editing, Supervision, Funding acquisition, Conceptualization.

## Declaration of generative AI and AI-assisted technologies in the writing process

During the preparation of this work, the authors used ChatGPT in order to check grammar for a better readership. After using this tool, the authors reviewed and edited the content as needed and take full responsibility for the content of the publication.

## DECLARATION OF COMPETING INTERESTS

The authors declare that they have no known competing financial interests or personal relationships that could have appeared to influence the work reported in this paper.
